# The Short Lipopeptides (C_10_)_2_-KKKK-NH_2_ and (C_12_)_2_-KKKK-NH_2_ Protect HaCaT Keratinocytes from Bacterial Damage Caused by *Staphylococcus aureus* Infection in a Co-Culture Model

**DOI:** 10.3390/antibiotics9120879

**Published:** 2020-12-08

**Authors:** Kirsten Reddersen, Katarzyna E. Greber, Izabela Korona-Glowniak, Cornelia Wiegand

**Affiliations:** 1 Klinik für Hautkrankheiten, Universitätsklinikum Jena, 07743 Jena, Germany; c.wiegand@med.uni-jena.de; 2 Physical Chemistry Department, Faculty of Pharmacy, Medical University of Gdansk, 80-416 Gdansk, Poland; katarzyna.greber@gumed.edu.pl; 3 Department of Pharmaceutical Microbiology, Medical University of Lublin, 20-093 Lublin, Poland; iza.glowniak@umlub.pl

**Keywords:** short synthetic antimicrobial lipopeptides, coculture model, HaCaT keratinocytes, *Staphylococcus aureus*, in vitro infection model, cell protection

## Abstract

The search for new antimicrobial strategies is of major importance since there is a growing resistance of both bacteria and fungi to existing antimicrobials. Lipopeptides are promising and potent antimicrobial compounds. For translation into clinically useful molecules, effectiveness of peptide treatment against human infections must be proved in complex in vitro wound models. The aim of this study was to examine if the synthesized short lipopeptides (C_10_)_2_-KKKK-NH_2_ and (C_12_)_2_-KKKK-NH_2_ can protect HaCaT keratinocytes from bacterial damage caused by *Staphylococcus aureus* infection in a coculture model. After 1 h, 24 h, and 48 h incubation, cellular ATP level and release of the cytotoxicity marker LDH as well as the proinflammatory cytokines interleukin-6 and interleukin-1α were measured. Infection of the keratinocytes resulted in strong bacterial damage of HaCaT cells along with low cellular ATP levels and high release of LDH, IL-6, and IL-1α after 24 h and 48 h. Incubation of the infected human keratinocytes with (C_10_)_2_-KKKK-NH_2_ and (C_12_)_2_-KKKK-NH_2_ resulted in protection of the keratinocytes from bacterial damage caused by *Staphylococcus aureus* infection with ATP, LDH, IL-6, and IL-1α levels comparable to the untreated control. Hence, both synthesized lipopeptides are promising candidates with high therapeutic potential in dermatology for the treatment of topical infections.

## 1. Introduction

*Staphylococcus aureus* (*S. aureus*) is one of the major causes of skin infections such as cellulitis, impetigo, furunculosis, or folliculitis in humans [1]. Each year, infections caused by *S. aureus* result in between 11 and 14 million outpatients and almost 500,000 hospitalizations in the United States [2]. *S. aureus* is further associated with delayed wound healing, and 71% of patients with a chronic wound carried this bacterium, with 30% of those identified as methicillin-resistant *S. aureus* (MRSA) [3,4]. The pathophysiology of *S. aureus* is multifactorial and involves a number of virulence factors. These extracellular and cell wall components are coordinately expressed depending on the different stages of infection, such as adhesion, host defense avoidance, growth, and bacterial spread [5]. Adhesion of *S. aureus* to host tissue is achieved by cell-wall-anchored proteins such as staphylococcal protein A (SpA), fibronectin-binding proteins A and B (FnbpA and FnbpB), collagen-binding protein, and clumping factor (Clf) A and B proteins [5]. *S. aureus* secretes a group of exoproteins such as exotoxins and enzymes, that convert host tissue into nutrients required for bacterial growth. The toxins of *S. aureus* can be divided into cytolytic toxins like α-toxin or Panton–Valentine leukocidin (PVL) that form ß-barrel pores in the plasma membrane followed by lysis of the target cells, and superantigene toxins which can induce immune-mediated damage like toxic shock syndrome. Additionally, specific virulence factors of *S. aureus* can affect the host immune system and thus promote the infection, e.g., staphylococcal superantigen-like protein-5 and protein-11 can inhibit neutrophil recruitment to sites of infection or SpA that can inhibit opsonization of the bacteria by immunoglobulin G [6]. Due to widespread use of antibiotics, strains of *S. aureus* have developed resistance to a variety of antibiotics like penicillin, vancomycin, cephalosporin, methicillin, and linezolid. All over the world, MRSA has become the most abundant resistant pathogen identified, e.g., in China, the proportion of hospital-acquired MRSA has reached 50.6% [7]. In the resistant strains, the effects of penicillin are abrogated by producing β-lactamase, and MRSA strains have acquired the *mec* gene that encodes penicillin-binding protein 2a, and the *fem* gene that provides resistance against several antibiotics [8].

Bacterial skin infections are counteracted by the innate and adaptive immune system. First defense of the host is achieved by immune response proteins like the complement system and transferrin that quickly activate a cascade reaction and trigger inflammatory responses [6]. Keratinocytes are the major cell type in the epidermis and are important in cutaneous immune response. After penetration of the epidermis by *S. aureus*, keratinocytes recognize the bacteria by pattern recognition receptors (PRRs) like TLR2 or NOD2. Signals from both receptors lead to activation of nuclear factor-κB (NF-κB) and mitogen-activated protein kinases (MAPK), transcription factors that activate keratinocytes to release and produce early-stage immune response proteins such as interleukin-1α (IL-1α) and interleukin-1β (IL-1β). These early signals activate the IL-1 receptors on keratinocytes and many other inflammatory cells, which results via activation of NF-κB in the production of several proinflammatory cytokines such as TNF-α, IL-6, IL-7, and IL-18 [6]. These proinflammatory cytokines also induce expression of neutrophil-attracting chemokines such as IL-8 to direct neutrophils to the site of infection. Keratinocyte activation also results in the production of antimicrobial peptides (AMPs), small, predominantly cationic proteins with bacteriostatic or bactericidal activity such as defensins and cathelicidins. These peptides have a high affinity to anionic surfaces, resulting in bacterial membrane damage followed by disruption of the ion gradient and bacterial death. In the adaptive immune response against *S. aureus* infection, T helper cells that produce IL-17 play a key role [9]. IL-17 promotes neutrophil recruitment and activation of IL-17 receptors, abundantly expressed on keratinocytes, that leads to production of antimicrobial peptides and increase of *S. aureus* bacterial clearance [10].

To promote healing in infected wounds, wound management aims at restoring a physiological milieu and combating bacterial growth. This can be achieved by antimicrobial treatment. Growing resistance of both bacteria and fungi to existing drugs is currently a worldwide concern [11]. Antibiotic resistance is associated with failure of clinical treatments, additional mortality, and increased health care costs [12]. For that reason, the search for new antimicrobial strategies is of major importance.

Antimicrobial lipopeptides are considered promising leads to develop novel antibiotics. They demonstrate potent antimicrobial activities against a broad spectrum of pathogens with a rapid mode of action. Additionally, they possess a different antimicrobial mechanism compared to most of the conventional antibiotics used today as well as a low tendency for development of drug resistance [12]. However, several candidates have relatively high cytotoxicity, hence a lot of attention has been paid to designing new molecules with optimal properties [13].

**Figure 1:**
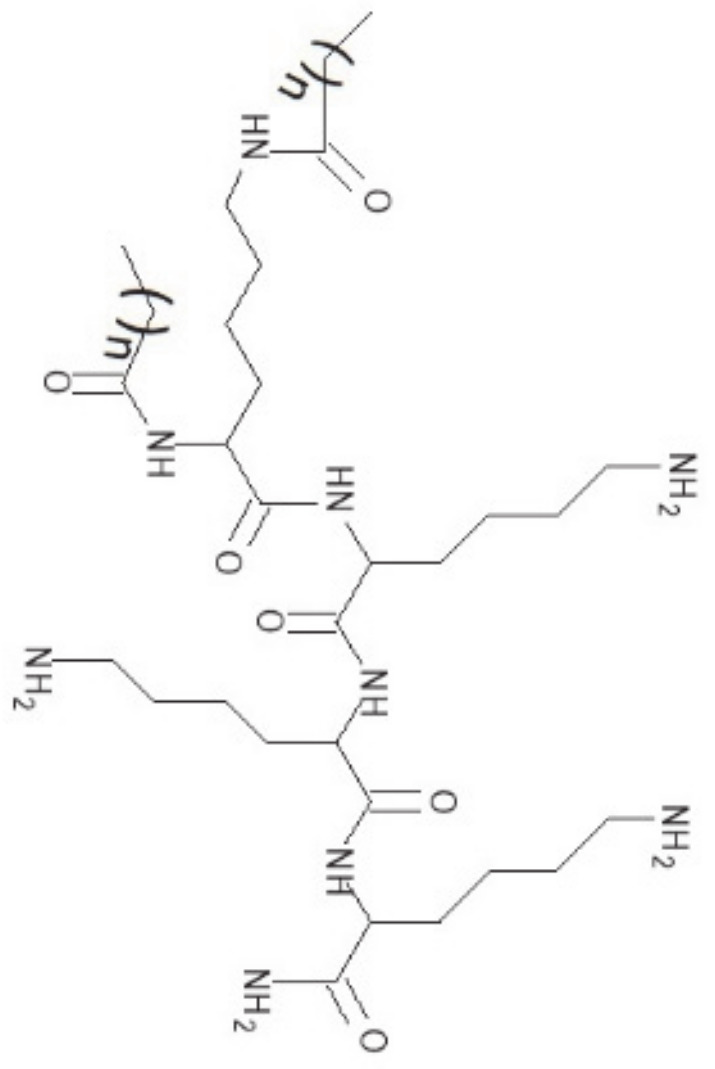
Molecular structure of the lipopeptides (C_10_)_2_-KKKK-NH_2_ (*n* = 8) and (C_12_)_2_-KKKK-NH_2_ (*n* = 10).

Two promising potential candidates are the short synthetic lipopeptides (C_10_)_2_-KKKK-NH_2_ and (C_12_)_2_-KKKK-NH_2_. These dialkylic lipopeptides with a positive net charge of +3 are composed of four lysine residues and two fatty acid chains attached to the α-amino and ε-amino moieties of the N-terminal lysine [14]. The examined lipopeptides differ in the length of the attached fatty acid chains, decanoic (capric) acid in (C_10_)_2_-KKKK-NH_2_, and dodecanoic (lauric) acid in (C_12_)_2_-KKKK-NH_2_ (Figure 1). They demonstrate a high antimicrobial activity against Gram-positive bacteria such as *Staphylococcus aureus*, *Staphylococcus epidermidis*, *Bacillus subtilis*, and *Enterococcus faecalis* and Gram-negative bacteria such as *Escherichia coli*, *Klebsiella pneumonia*, and *Pseudomonas aeruginosa*. Additionally, a moderate antifungal activity against *Candida albicans*, *Candida tropicalis*, and *Aspergillus niger* was determined [14]. They are also effective against methicillin-resistant *Staphylococcus aureus* (MRSA) [15]. Both lipopeptides display low cytotoxicity on HaCaT keratinocytes with IC_50_ values a few times higher compared to minimum inhibitory concentrations (MIC) obtained for the tested bacteria, indicating a therapeutic potential in dermatology for the treatment of topical infections [13].

However, for translation of these promising antimicrobial peptides into clinically useful molecules, the effectiveness of peptide treatment against human skin infections must be proved in more complex in vitro wound models. Therefore, the aim of this study was to examine if the synthesized lipopeptides (C_10_)_2_-KKKK-NH_2_ and (C_12_)_2_-KKKK-NH_2_ can protect HaCaT keratinocytes from bacterial damage caused by *S. aureus* infection in a coculture model.

## 2. Results

In a coculture model of HaCaT keratinocytes and *S. aureus,* which serves as an in vitro model for infected wounds [16], the antimicrobial and cytocompatibility properties of the ultrashort lipopeptides (C_10_)_2_-KKKK-NH_2_ and (C_12_)_2_-KKKK-NH_2_ were examined.

**Figure 2:**
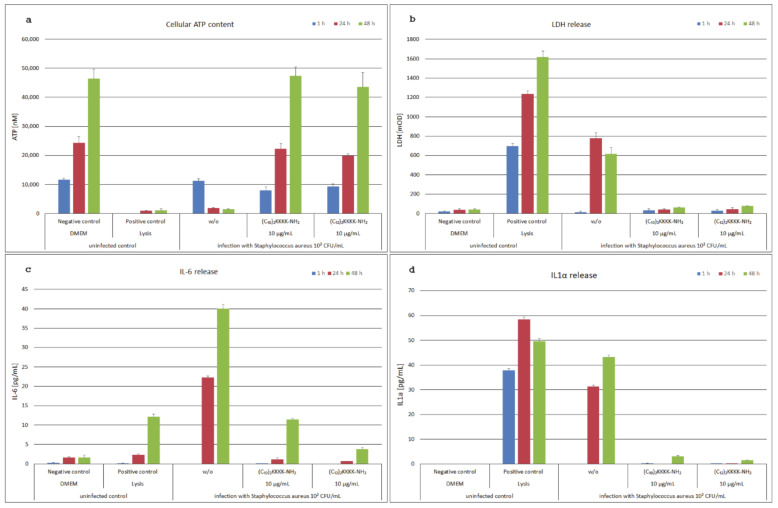
The coculture model of HaCaT keratinocytes infected with *Staphylococcus aureus* during incubation for 1 h, 24 h, and 48 h with (C_10_)_2_-KKKK-NH_2_ and (C_12_)_2_-KKKK-NH_2_ compared to the uninfected controls. In panel (**a**), the cellular ATP content of the *S. aureus*-infected samples showed low viability comparable to the positive control of lysed cells. Treatment with (C_10_)_2_-KKKK-NH_2_ and (C_12_)_2_-KKKK-NH_2_ at 10 µg/mL protected the HaCaT keratinocytes from bacterial damage and showed viability comparable to the DMEM-treated negative control. In panel (**b**), the release of the toxicity marker LDH was highest in the lysed cells of the positive control, but it was also considerably enhanced after 24 h and 48 h infection with *S. aureus.* During incubation of the infected keratinocytes with (C_10_)_2_-KKKK-NH_2_ and (C_12_)_2_-KKKK-NH_2_, the release of the cytotoxicity marker LDH was prevented. Panel (**c**) shows a considerable increase of the release of the proinflammatory cytokine IL-6 by infection of HaCaT keratinocytes with *S. aureus* after 24 h and 48 h. This was prevented by incubation of the infected model with (C_10_)_2_-KKKK-NH_2_ and (C_12_)_2_-KKKK-NH_2_. Similar effects were shown in panel (**d**) for the release of the proinflammatory cytokine IL-1α.

In Figure 2a, the cellular ATP content of the HaCaT keratinocytes after 1 h, 24 h, and 48 h incubation is shown. In the untreated, healthy (negative) control, the ATP content was doubling each 24 h, whereas in the cytotoxicity (positive) control ATP levels were low. Infection with *S. aureus* resulted in severe damage of the HaCaT keratinocytes with low cell viability after 24 h and 48 h. Treatment of infected cells with the lipopeptides (C_10_)_2_-KKKK-NH_2_ and (C_12_)_2_-KKKK-NH_2_ prevented bacterial damage to the HaCaT keratinocytes following *S. aureus* infection and restored cell viability to untreated control levels.

Similar results were obtained by measuring the cytotoxicity marker lactate dehydrogenase (LDH) in the cell culture supernatants (Figure 2b). The highest LDH levels were determined in the cytotoxicity control, whereas LDH levels in the untreated control were low at all incubation times. Infection of the HaCaT keratinocytes with *S. aureus* resulted in cellular damage and, consequently, a considerable increase of LDH in the supernatants after 24 h and 48 h. LDH levels of the infected models incubated with the lipopeptides were low after 24 h and 48 h, indicating a protection of the keratinocytes from bacterial damage by both examined lipopeptides after *S. aureus* infection.

The levels of the proinflammatory cytokine IL-6 in the cell culture supernatants were highest in the *S. aureus*-infected keratinocytes. After 1 h incubation, there was no detectable IL-6 release, but it considerably increased after 24 h and even more after 48 h (Figure 2c). IL-6 levels in the untreated control were low, in the cytotoxicity control comparable low after 24 h and slightly increased after 48 h. Treatment of the infected keratinocytes with the lipopeptides resulted in low IL-6 levels, especially after 24 h incubation, comparable to the untreated control.

IL-1α, a proinflammatory cytokine and early marker of cellular damage, was detected in the cytotoxicity control at all incubation times, whereas it was not detectable in the supernatants of the untreated control (Figure 2d). Infection of the HaCaT keratinocytes with *S. aureus* resulted in a considerable IL-1α release after 24 h and 48 h incubation. Addition of (C_10_)_2_-KKKK-NH_2_ and (C_12_)_2_-KKKK-NH_2_ prevented cellular damage with low IL-1α levels after 24 h and 48 h incubation. 

## 3. Discussion

In the search for novel antimicrobials to combat increasing resistance of bacteria and fungi, antimicrobial peptides play an important role. In this study, the synthesized short lipopeptides (C_10_)_2_-KKKK-NH_2_ and (C_12_)_2_-KKKK-NH_2_ were examined for its ability to protect HaCaT keratinocytes from bacterial damage by *S. aureus* in the in vitro coculture model.

*S. aureus* is a universally occurring Gram-positive pathogen. It plays a significant role in cutaneous and systemic infections and is reported to colonize the anterior nares in 20–40% of healthy individuals [17]. Presence of *S. aureus* is problematic in several types of infections such as traumatic, surgical, and burn wound infections [16]. During infection of human keratinocytes with *S. aureus*, cells are permeabilized and killed by low concentrations of α-toxin [18]. This channel-forming protein induces keratinocyte necrosis. As a result, a considerable release of the cytotoxicity marker LDH and decrease of cellular ATP level follows, which was visible in our study 24 h and 48 h after infection with *S. aureus*. In this coculture model, the inoculum concentration of *S. aureus* was relatively low, enabling the keratinocytes to exhibit an inflammatory response and avoiding rapid killing of the human cells [16]. This model has already been successfully used to demonstrate the protective effect of the disinfectant polyhexamethylene biguanide (PHMB) on keratinocytes against damage caused by bacterial infection with *S. aureus* [16]. The keratinocytes secreted an increasing amount of the pro-inflammatory cytokines IL-6 and IL-1α 24 h and 48 h after infection with *S. aureus*. Cytokines, small soluble proteins, play a major role in regulation of acute and chronic inflammation. Different cells secrete these cytokines that act via a complex signal cascade. This includes keratinocytes, macrophages, monocytes, T cells, and endothelial cells [19]. The increased secretion of the proinflammatory cytokines IL-6 and IL-1α as host defense after infection with *S. aureus* was also observed in 2D and 3D cell models [16,19,20,21].

Lipopeptides display high antimicrobial activities against different pathogens. They act rapidly via different modes of action compared to most conventional antibiotics, so the tendency to develop drug resistance is low [12]. Lipopeptides consist of a hydrophilic peptide and a hydrophobic fatty acyl chain. The positive net charge and the amphipathic structure are crucial for the cytolytic action of these substances [22]. Bacterial cell membranes are perturbed by lipopeptides causing the death of the bacteria [12]. Two different mechanisms are relevant for binding of lipopeptides to lipid membranes: electrostatic interaction between the peptide and the lipid phosphate and hydrophobic interactions with the acyl chains [23]. With regard to antimicrobial research, in recent years, the focus shifted from natural lipopeptides produced in bacteria and fungi to synthesis of mimics with improved properties, such as lower production costs and higher stability.

(C_10_)_2_-KKKK-NH_2_ and (C_12_)_2_-KKKK-NH_2_, two synthesized short lipopeptides, exhibit a high antimicrobial potential against a diversity of Gram-positive and Gram-negative bacteria and a moderate activity against fungi [13,14,15]. Additionally, they demonstrated good cytocompatibility on HaCaT keratinocytes. Therefore, both lipopeptides were studied in a coculture model of HaCaT keratinocytes and *S. aureus*, which serves as an in vitro model for skin infections. After 24 h and 48 h treatment of the infected model with the lipopeptides, cellular ATP levels were comparable to the untreated control and only marginal amounts of the cytotoxicity marker LDH were detected in the supernatants. These data indicate that the addition of these lipopeptides to the infected keratinocytes resulted in rapid antibacterial action and therefore in protection of the human keratinocytes from bacterial damage. Additionally, the proinflammatory reaction of the keratinocytes caused by *S. aureus* infection was prevented by treatment with the lipopeptides. Greber et al., (2020) assumed a membrane-damaging mechanism of action of the tested lipopeptides after incubation with *S. aureus* that was supported by morphological changes observed by scanning electron microscopy and fast bactericidal action measured in time-kill analyses [15]. Presumably, the lipopeptides interact with acidic phospholipids in the microbial membranes followed by bacterial cell death and feature a much lower affinity to neutral phospholipids in the keratinocyte cell membranes. In the coculture infection model, the lipopeptides prevented the release of staphylococcal toxins that provoke an inflammatory response of the keratinocytes through their fast-acting bactericidal activity. In this way, the release of the proinflammatory cytokines IL-6 and IL-1α was inhibited after treatment of the infected keratinocytes with the lipopeptides. The results of the protective, anti-inflammatory effect of the lipopeptides in our coculture model are in concordance with a study of Wu et al., (2020), who examined the anti-inflammatory effect of the synthesized antimicrobial peptide bombine-like peptide 7 (BLP-7) in a coculture model of normal human epidermal keratinocytes (NHEK) and *Propionibacterium acnes.* They also found a significantly reduced production of proinflammatory cytokines such as interleukine-8 (IL-8) and granulocyte-macrophage colony-stimulating factor (GM-CSF) after treatment of the infected keratinocytes with the peptide [24].

In our study, no differences were observed between the protective effects of (C_10_)_2_-KKKK-NH_2_ and (C_12_)_2_-KKKK-NH_2_. Hence, both synthesized lipopeptides are promising candidates with high therapeutic potential in wound healing for the treatment of topical infections.

## 4. Materials and Methods

### 4.1. Lipopeptide Synthesis

The lipopeptides (C_10_)_2_-KKKK-NH_2_ (N-α-decanoyl-N-ε-decanoyl-Lys-Lys-Lys-Lys-NH_2_) and (C_12_)_2_-KKKK-NH_2_ (N-α-dodecanoyl-N-ε-dodecanoyl-Lys-Lys-Lys-Lys-NH_2_) were manually synthesized according to standard solid-phase Fmoc-protocol as described in [15]. Lipopeptides were purified using high-performance liquid chromatography (RP-HPLC) and identity of the lipopeptides verified by matrix-assisted laser desorption time-of-flight spectrometry (MALDI-TOF, Birfex III, Bruker, Germany) [15]. Purified lipopeptides with confirmed identity were freeze-dried and stored as dry powder at −20 °C.

### 4.2. Coculture Model

The coculture model of HaCaT keratinocytes infected with *Staphylococcus aureus* was carried out according to Wiegand et al., 2009 [16]. *S. aureus* ATCC 6538 was purchased from the DSMZ (Deutsche Sammlung von Mikroorganismen und Zellkulturen, Braunschweig, Germany). (C_10_)_2_-KKKK-NH_2_ and (C_12_)_2_-KKKK-NH_2_ were dissolved in DMSO (Sigma-Aldrich, Germany) at 10 mg/mL, sterilized by passage through a 0.2 µm filter, and diluted in Dulbecco’s modified Eagle’s medium (DMEM, Promocell, Germany) to a final concentration of 20 µg/mL. The concentration of the lipopeptides, which is additionally halved in the incubation model, was chosen according to previous studies of both lipopeptides on antimicrobial activity [14] and cytotoxicity on HaCaT keratinocytes [13]. Overnight cultures of *Staphylococcus aureus* (ATCC 6538, DSMZ, Germany) were adjusted to a concentration of 10^2^ CFU/mL in tryptone soya broth (TSB, Oxoid, Germany). HaCaT keratinocytes (a gift from Prof. Dr. N.E. Fusenig, DKFZ, Germany), seeded for 48 h at a density of 40,000 cells/cm^2^ into 96-well microtiter plates (Greiner, Germany), were preincubated with the bacteria for 1 h before solutions of the lipopeptides and controls were respectively added. Details of methodology are described in Wiegand et al., 2009 [16]. After 1 h, 24 h, and 48 h incubation, cell proliferation was determined using a luminometric ATP assay according to manufacturer instructions (ATPLite™, Perkin Elmer, Waltham, MA, USA). Cell culture supernatants were obtained and LDH release was determined according to manufacturer instructions by colorimetric assay (Roche Diagnostic GmbH, Mannheim, Germany). As positive damage control, lysis of the keratinocytes was induced 45 min prior to the respective time of investigation by adding mammalian cell lysis buffer (ATPLite™, Perkin Elmer, Waltham, MA, USA). Aliquots of the supernatants were kept at −20 °C until determination of cytokine levels according to manufacturer instructions (human IL-6 ELISA, Mabtech, Sweden; human IL-1a ELISA, R&D Systems, MN, USA).

Experiments were performed in duplicate and each sample was measured in four replicates. All values presented are expressed as means ± SD. One-way analysis of variance was carried out to determine statistical significances (Microsoft^®^ Excel 2016). Differences were considered statistically significant at a level of *p* < 0.05.
